# Successful Endovascular Management of Recurrent Hemoptysis due to Multiple Rasmussen Aneurysms in a Case of Pulmonary Tuberculosis: A Rare Case Scenario

**DOI:** 10.15388/Amed.2024.31.1.25

**Published:** 2024-02-27

**Authors:** Shritik Devkota, Harish Bhujade, Abhiman Baloji, Nidhi Prabhakar, Atul Saroch, Ujjwal Gorsi

**Affiliations:** 1 Department of Radiodiagnosis and Imaging, Post Graduate Institute of Medical Education & Research, Chandigarh, India; 2 Department of Internal Medicine, Post Graduate Institute of Medical Education & Research, Chandigarh, India

**Keywords:** Rasmussen aneurysm, pulmonary tuberculosis, pulmonary artery pseudoaneurysm, coil embolization, Rasmusseno aneurizma, plaučių tuberkuliozė, plaučių arterija

## Abstract

**Background:**

Hemoptysis is defined as coughing out of blood. Pulmonary tuberculosis is the most common cause of hemoptysis in tuberculosis-endemic countries like India. Rasmussen aneurysm is a pseudoaneurysm arising from the pulmonary artery adjacent to or within a tuberculous cavity. Chest radiographs, chest computed tomography angiography (CTA), and digital subtraction angiography (DSA) are the imaging tools for evaluating a case of hemoptysis.

**Case:**

A 32-year-old man with a history of pulmonary tuberculosis presented with complaints of recurrent hemoptysis. On imaging evaluation, multiple pulmonary artery pseudoaneurysms were seen in the left lung. The patient was shifted to the DSA lab and the pseudoaneurysms were subsequently treated by endovascular coil embolization. Hemoptysis resolved following the procedure and the patient was again started on anti-tubercular therapy.

**Conclusion:**

Endovascular coiling is minimally invasive, safe, and effective management of multiple Rasmussen aneurysms for preventing possible torrential blood loss and unfortunate death.

## Abstract (it)

**Įvadas:**

Hemoptizė yra kosulys su krauju. Endeminėse šalyse, pavyzdžiui, Indijoje, plaučių tuberkuliozė yra dažniausia hemoptizės priežastis. Rasmusseno aneurizma yra pseudoaneurizma, atsirandanti iš plaučių arterijos, esančios šalia tuberkuliozinės ertmės arba jos viduje. Krūtinės ląstos rentgenogramos, krūtinės ląstos kompiuterinės tomografijos angiografija (KTA) ir skaitmeninė subtrakcinė angiografija (DSA) – tai vaizdinimo priemonės hemoptizės atvejui įvertinti.

**Atvejis:**

32 metų vyras, sirgęs plaučių tuberkulioze, skundėsi pasikartojančia hemoptize. Atlikus vaizdų vertinimą, kairiajame plautyje buvo matomos daugybinės plaučių arterijos pseudoaneurizmos. Pacientas buvo perkeltas į DSA laboratoriją, o vėliau pseudoaneurizmos gydytos endovaskuline spiraline embolizacija. Po procedūros hemoptizė išnyko ir pacientui vėl buvo pradėtas antituberkuliozinis gydymas.

**Išvados:**

Endovaskulinė embolizacija yra minimaliai invazinis, saugus ir veiksmingas daugybinių Rasmusseno aneurizmų gydymas, siekiant išvengti galimo didelio kraujo netekimo ir nelaimingos mirties.

## Introduction

Hemoptysis is defined as coughing out of blood [1-5]. There are many definitions cited in literature for grading hemoptysis as massive or life threatening. A consistent definition for “massive haemoptysis” remains elusive, with the literature offering a wide range of thresholds based on expectorated blood volume [1-4]. 100 ml is the smallest volume of hemoptysis in 24 hours that has been described in the literature as endangering the patient’s life [2].

There are several causes of hemoptysis such as pulmonary tuberculosis (both primary and post-primary), malignancies, other infections, bronchiectasis, pulmonary embolism, heart failure, iatrogenic (anticoagulants, bronchoscopy, biopsy), arteriovenous malformation, trauma, foreign body, bronchitis and vasculitis [6-10]. Hemoptysis may be associated with pulmonary tuberculosis (PTB) in about 8–10% of all cases. The sources of bleed in case of pulmonary tuberculosis can be either due to Rasmussen aneurysm caused by destructive cavitary lung changes, bronchial artery hypertrophy or erosion of a calcified granulomatous lymph node into the airway [6-10].

Rasmussen aneurysm is a pseudoaneurysm arising from the pulmonary artery adjacent to or within a tuberculous cavity [11-13]. Rasmussen’s aneurysm arises from a sequential weakening of the pulmonary artery wall. Granulation tissue replaces the structurally supportive adventitia and media layers, ultimately being replaced by less robust fibrin. This progressive deterioration renders the wall thin and susceptible to pseudoaneurysm formation [11,14-18].

Pseudoaneurysms, lacking a complete vascular wall, are prone to haemorrhage within the lung parenchyma or bronchial tree. Mycobacterial tuberculosis isn’t the sole infectious cause of pulmonary artery pseudoaneurysms; fungal and bacterial pneumonia can also lead to them. Non-infectious mechanisms, including tumor invasion, vasculitis, radiation, trauma, and iatrogenic procedures, are also prevalent [6,19,20].

Chest X-ray guides initial hemoptysis evaluation, but chest CT angiography remains the gold standard for definitive diagnosis. Detailed imaging evaluation followed by timely intervention is needed for mitigating the risk of increased morbidity and mortality related to Rasmussen aneurysm [5-7,9,10,19,20].

Hemoptysis primarily leads to mortality through asphyxiation rather than exsanguination [7,12]. The cornerstone of hemoptysis management involves hemodynamic resuscitation and airway patency followed by targeted therapy addressing the underlying etiology to minimize mortality risk [7,9,10,12].

Hemoptysis secondary to Rasmussen aneurysm is a rare entity. Furthermore, only few case reports are available in the literature regarding hemoptysis secondary to multiple Rasmussen aneurysm [12,21-24]. We aimed to share our experience in successfully managing a rare and challenging case of multiple Rasmussen’s aneurysms in a patient with known pulmonary tuberculosis with endovascular embolization.

## Case report

A 32-year-old male presented to the emergency department with recurrent episodes of mild hemoptysis (volume of ~20–25 ml/episode) and intermittent low-grade fever for last 6 months. He had previous history of pulmonary tuberculosis one year back for which he had completed 6 months of antitubercular therapy (ATT). He had no other comorbidities. Vitals were stable at the time of presentation (Pulse rate – 76/min, blood pressure – 116/78 mm of Hg, respiratory rate – 16/min, oxygen saturation – 97% and normal general survey).

**Fig. 1:**
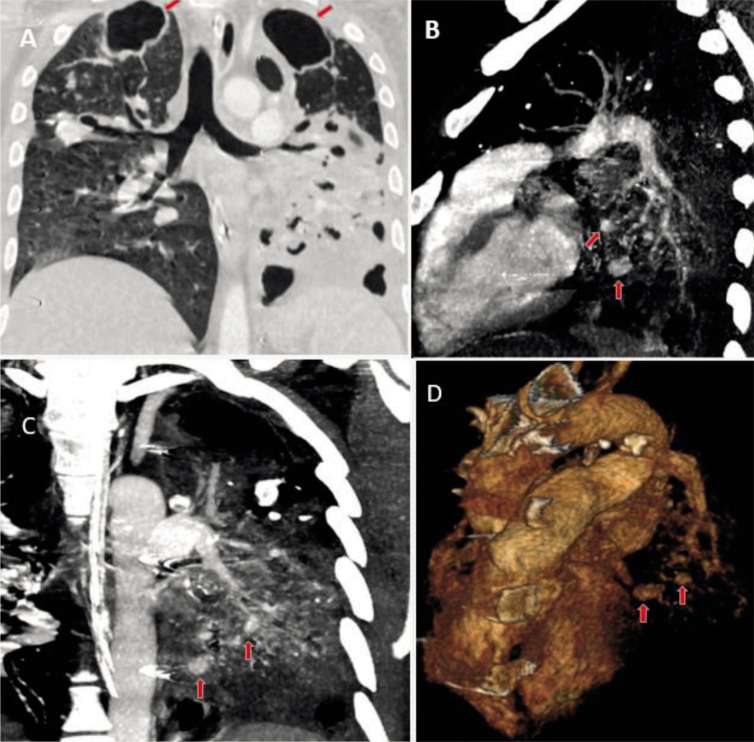
Chest CT angiography (CTA). Lung window (A) showing cavitations in bilateral upper lobes (red arrows) with destroyed lingula and left lower lobe. Maximum intensity projection (MIP) sagittal (B), coronal (C) and 3D virtual (D) reconstructions showing contrast filled outpouchings (red arrows) arising from branches of left pulmonary artery.

Sputum Gene-xpert was positive for Mycobacterium tuberculosis. He had low Hb level (~10.5 gm/dL). CTA (Figure 1) was done which revealed multiple contrast-filled outpouchings from branches of left pulmonary artery adjacent to fibrocavitary and fibro-bronchiectatic changes.

**Fig. 2:**
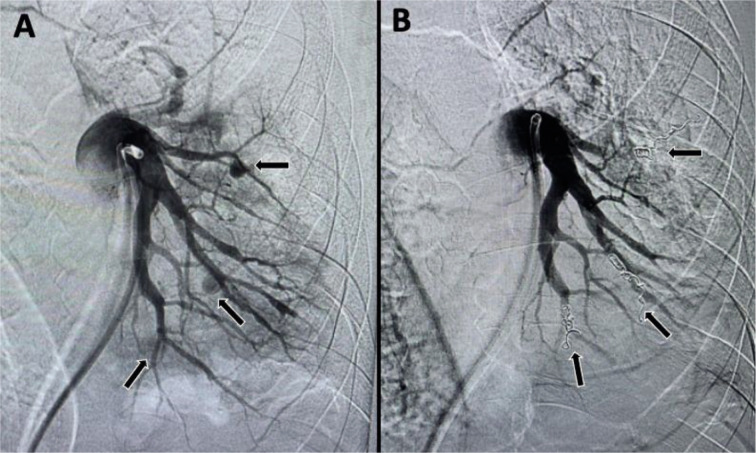
Digital subtraction angiography (DSA). Left pulmonary artery run (A) showing three contrast filled outpouchings (black arrows) from its branches. Final left pulmonary artery run (B) showing coil mass in situ with nonopacification of the previous outpouchings suggesting successful embolization (black arrows).

Thereafter, patient was shifted for pulmonary digital subtraction angiography (DSA). Informed consent was taken for the procedure. Using right common femoral venous access, left pulmonary angiogram (Figure 2A) was taken which showed three contrast-filled outpouchings from branches of left pulmonary artery. Superselective cannulation of each branch supplying the pseudoaneurysm was done using the Progreat microcatheter (Terumo medical corporation, New Jersey). The branch supplying each pseudoaneurysm was embolized using two 5 mm x 14 cm and one 4 mm x 14 cm Nester microcoils (Cook Medical, Bloomingtom, USA). Post-embolization, there was complete non-opacification of all pseudoaneurysms (Figure 2B). Hemoptysis resolved following the procedure. Patient was again started on multi-drug resistant ATT regimen.

## Discussion

Pulmonary tuberculosis can present in varying spectrum extending from insidious systemic and respiratory symptoms to hemoptysis and multisystemic dissemination. Important causes of hemoptysis in pulmonary tuberculosis include bleeding from cavity wall, Rasmussen aneurysm, and bronchial artery hypertrophy [5,6,11,12,15,16]. Rasmussen aneurysm in itself is a rare entity, while literature regarding multiple Rasmussen aneurysms in same patient in same lung are scarce [12,21-24].

The term “Rasmussen” originates from the work of Fritz Valdemar Rasmussen et al., who provided a comprehensive pathological analysis of 11 cases in 1868 [25]. Rasmussen’s aneurysm manifests as a sequela of a progressive and detrimental cascade within the pulmonary arterial wall. This deleterious process commences with the insidious substitution of the structurally robust adventitia and media layers by less resilient granulation tissue. This initial compromise is ultimately compounded by the further supplantation of the granulation tissue with the markedly less robust fibrin. The culmination of this pathological odyssey is a demonstrably thinned and structurally compromised arterial wall, rendering it exceedingly susceptible to the formation of a pseudoaneurysm [5,6, 11,14-18].

Extensive clinical, laboratory and imaging evaluation is needed in patients presenting with hemoptysis. Patients’ symptomatology, history of comorbidities and prior ATT are critical when investigating a case of prior pulmonary tuberculosis [5,6,9,10,15]. Chest radiograph, chest CTA and DSA are the imaging tools. Chest radiograph is predominantly used as initial screening tool to have an overall gross idea about the disease. Chest X-ray can show cavitations, bronchiectasis, consolidations in such cases of tuberculosis [9,10,26]. Contrast enhanced CT (CECT) or chest CTA can further help us in knowing the culprit for hemoptysis in addition to the severity of parenchymal disease. On CTA, Rasmussen aneurysm is seen as a focal outpouching arising from pulmonary artery branches. The outpouching may show irregular wall. Surrounding contained hematoma may also be observed [5,6,19,20]. DSA serves as both diagnostic and interventional tool for management of Rasmussen aneurysm [5-7,16,17,19,20,22,23]. Furthermore, bronchoscopy serves a pivotal role in the management of hemoptysis by facilitating the precise localization of the bleeding site. This localization capability allows for the targeted application of therapeutic interventions aimed at achieving definitive haemostasis [9, 26-28].

Hemodynamic and airway stabilization are the foremost considerations when managing a case of hemoptysis [7,9,15,26,27]. Patients’ vitals are continuously monitored, and oxygenation is provided. Massive hemoptysis may even require endotracheal intubation for securing adequate respiratory gas exchange [9, 26-28]. Following the initial stabilization of vital signs, a comprehensive imaging workup is then undertaken. This detailed diagnostic evaluation aims to identify the underlying etiology responsible for the hemoptysis event. Only after a definitive diagnosis is established, targeted therapeutic interventions can be implemented to address the root cause and definitively control bleeding.

The vascular cause for hemoptysis is primarily treated by endovascular route. Endovascular management for Rasmussen aneurysm includes coil, plug or glue embolization [7-9,22,23,28]. In index case, microcoils were used as there is better control and precision compared to endovascular glue embolization. The side-branch anchor technique was used to place the microcoils to avoid the distal migration. For larger calibre vessels, vascular plug may be used. The microcoil is more cost-effective than vascular plug. Therefore, careful planning and choice of embolizing agent should be done to get desired outcome and less complications considering the cost of the embolization agents. This case highlights the usefulness of cross-sectional imaging for the diagnosis and planning of embolization. Coil embolization can be considered when there are multiple Rasmussen aneurysms without major complications.

## Conclusion

The index case emphasizes that hemoptysis may occur due to multiple Rasmussen aneurysms in a patient with post-primary tuberculosis. Careful evaluation of chest CTA helps to localize multiple aneurysms and is also beneficial for planning of the therapy. Endovascular coiling is a minimally invasive, safe and effective method for treatment of multiple Rasmussen aneurysms.
